# The Human Genes That Link Middle-Age Comorbidities And Alzheimer’s Disease

**DOI:** 10.1093/geroni/igab046.2424

**Published:** 2021-12-17

**Authors:** Shin Murakami

**Affiliations:** Touro University California, Vallejo, California, United States

## Abstract

Advancements in biomedical research have identified the genes influencing life spans, stress resistance and age-related diseases, including Alzheimer’s disease. Stress resistance includes resistance to multiple forms of stress, pathogens and toxic beta-amyloid which is tightly associated with Alzheimer’s disease. We have investigated 431 human genes that are associated with co-morbidities (Vahdati Nia et al, 2017; Le et al., 2020). Those genes are involved in lipid metabolism, hemostasis, hemostasis, neuroendocrine and immune functions. The genes are relevant to middle-life health. We explore a wide variety of co-morbidities that could happen in middle to late life. I will give a brief review of increased stress resistance, and genetic markers associated with co-morbidities. I will discuss how the studies may benefit to fight against COVID-19. References: 1. Vahdati Nia B, Kang C, Tran MG, Lee D, Murakami, S. (2017) Front. Genet. 8:55. doi: 10.3389/fgene.2017.00055 2. Le D, Crouch N, Villanueva A, Phong Ta, Dmitriyev R, Tunzi M, and Murakami S. (2020) Journal of Neurology and Experimental Neuroscience. 6;S1.

